# Supplementary figures and images for: Aberrant repair initiated by the adenine-DNA glycosylase does not play a role in UV-induced mutagenesis in Escherichia coli
Source: PeerJ. 2018 Dec 5;6:e6029. doi: 10.7717/peerj.6029 (PMC6286661; doi:10.7717/peerj.6029)

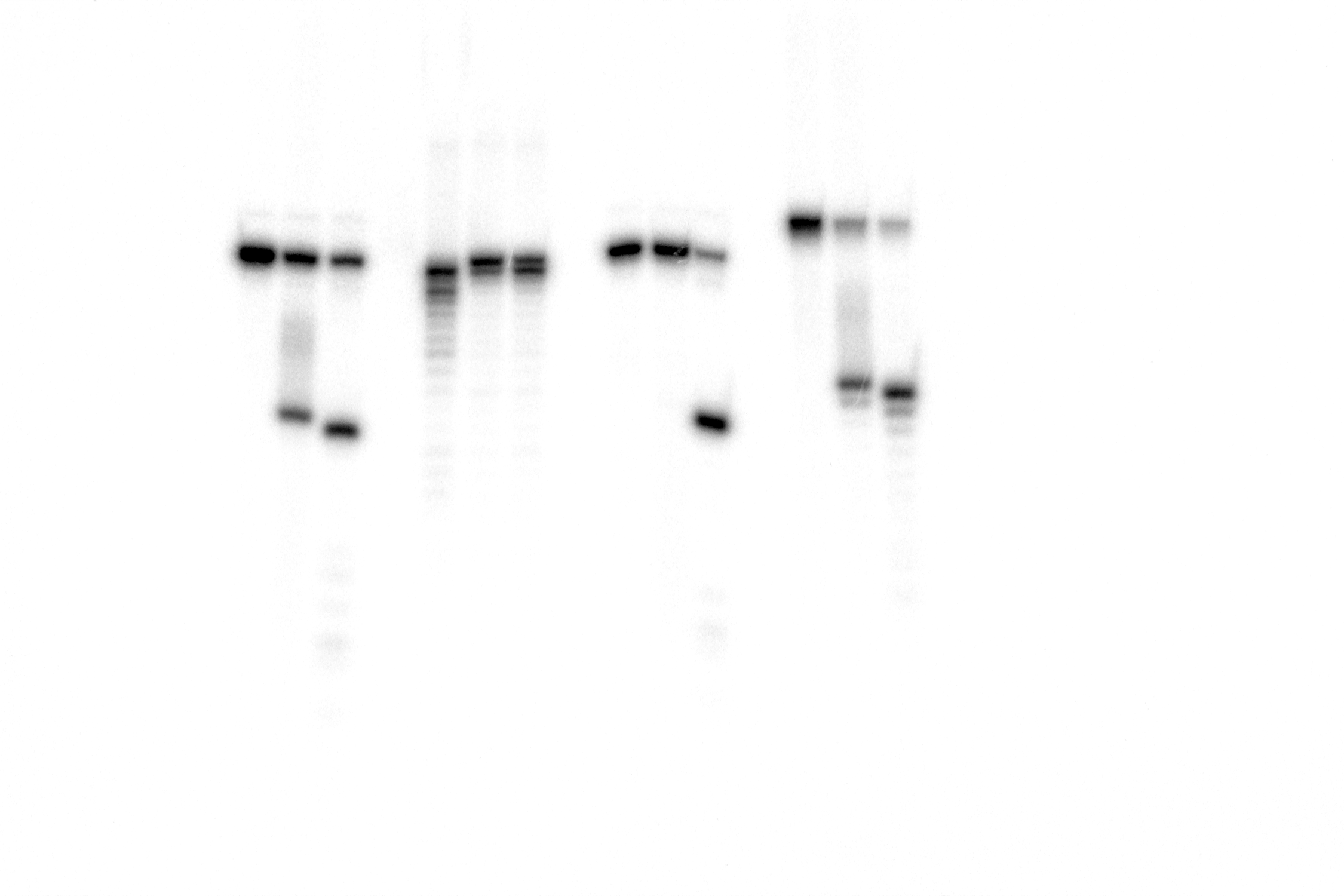

Supplement: Supplemental Information 1 — Lanes 1, 7 and 10, control non-treated oligonucleotides; lanes 2–3 and 8–9, 24 mer AA•T=T and AA•T-T duplexes incubated either with T4-PDG or UVDE; lanes 11 and 12, 30 mer AA • T=T duplex incubated with T4-PDG and UVDE, respectively; lanes 4–6, 24 mer regular AA•TT duplex incubated with MtbXth, a 3′-5′ exonuclease, to generate size markers. For details see Fig. 1 in the manuscript. [file peerj-06-6029-s001.png]

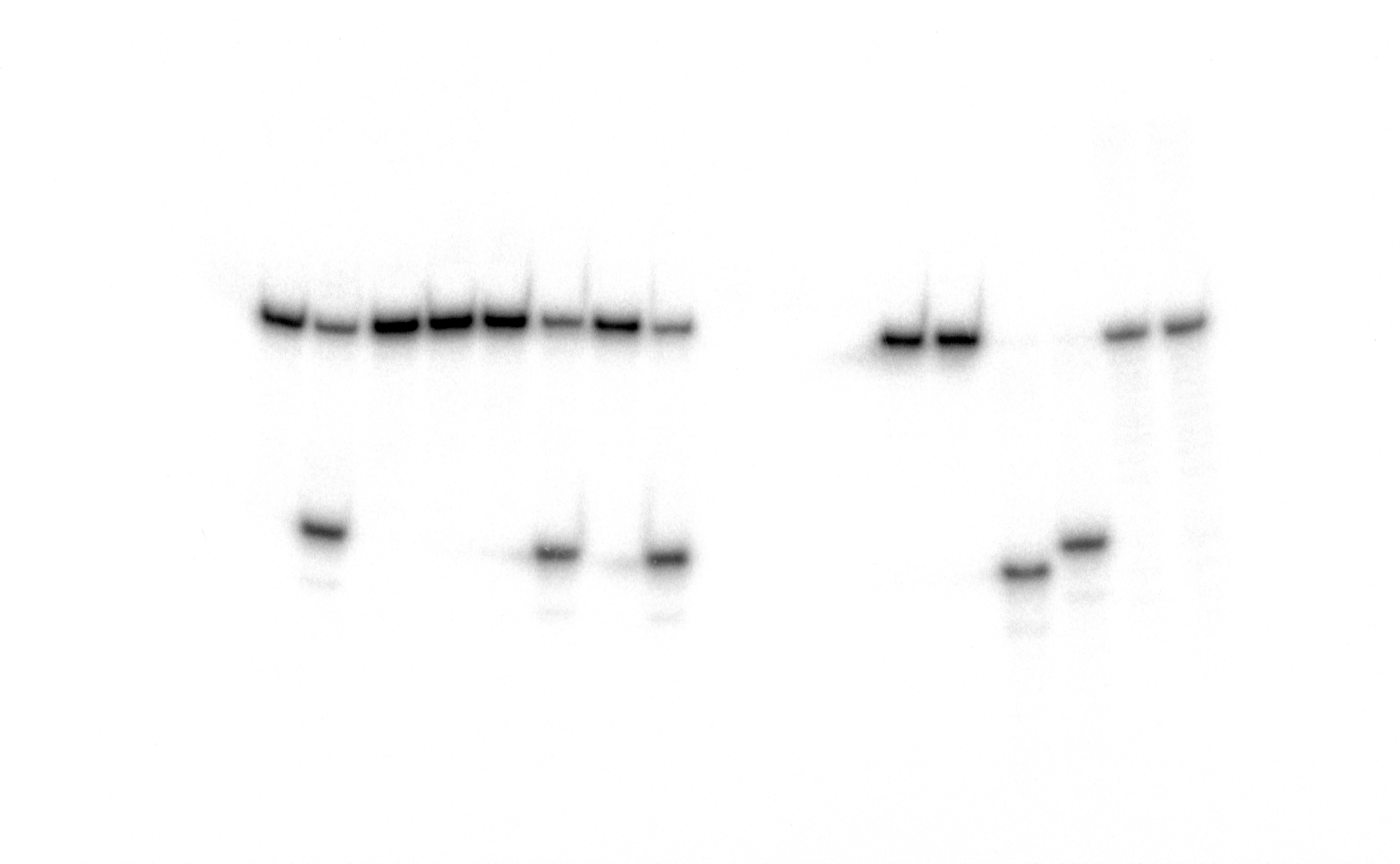

Supplement: Supplemental Information 2 — Lanes 1–10, 24 mer duplexes incubated or not with MutY; lanes 11–14, 24 mer single-stranded oligonucleotides containing single Uracil residue, incubated or not with Ung to generate size markers. For details see Fig. 2 in the manuscript. [file peerj-06-6029-s002.png]

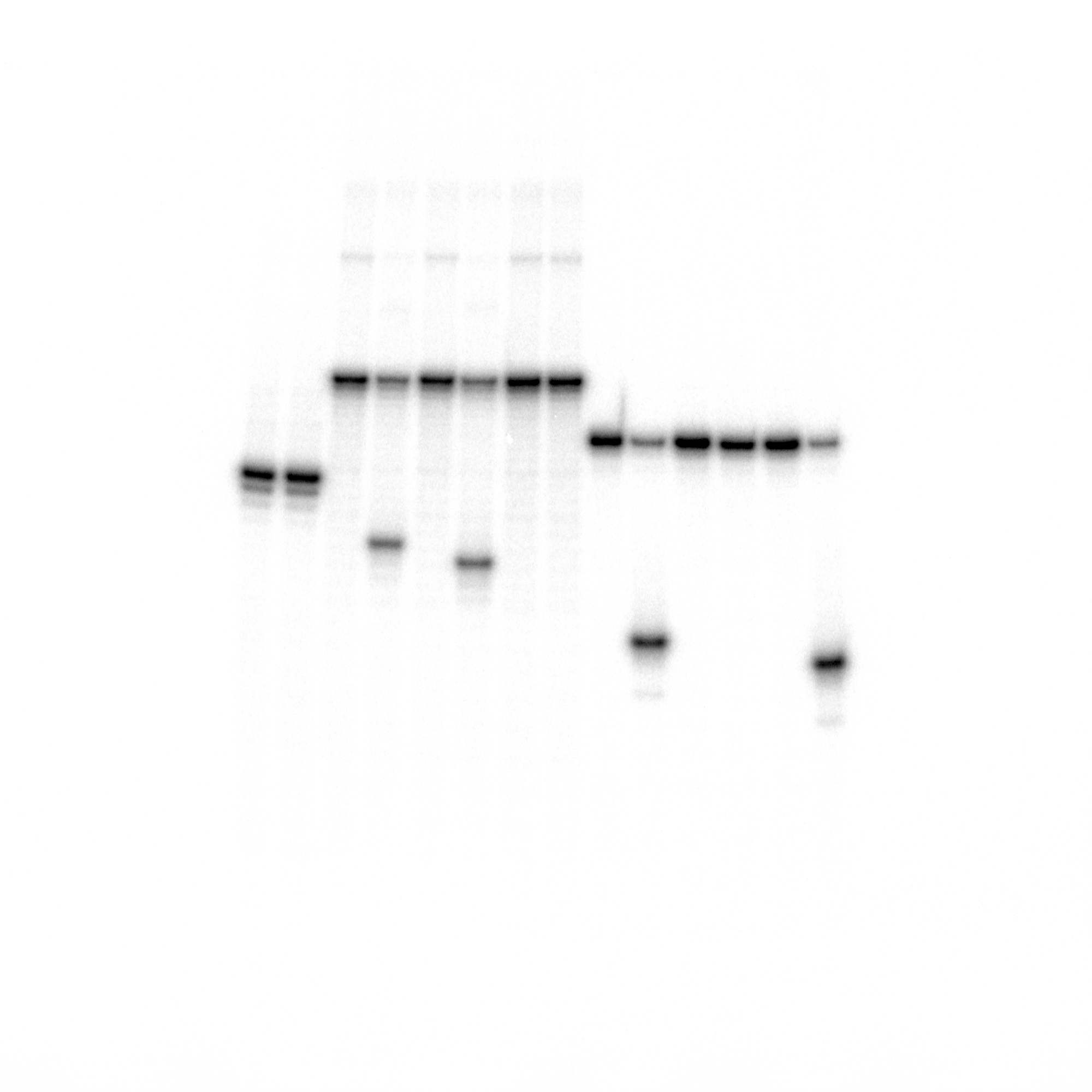

Supplement: Supplemental Information 3 — Lanes 1, 3 and 5, control non-treated 30 mer oligonucleotides; lanes 2, 4 and 6, 30 mer duplexes incubated with MutY; Lanes 7, 9 and 11, control non-treated 24 mer duplexes; lanes 8, 10 and 12, 24 mer duplexes incubated with MutY. For details see Fig. 3 in the manuscript. [file peerj-06-6029-s003.png]

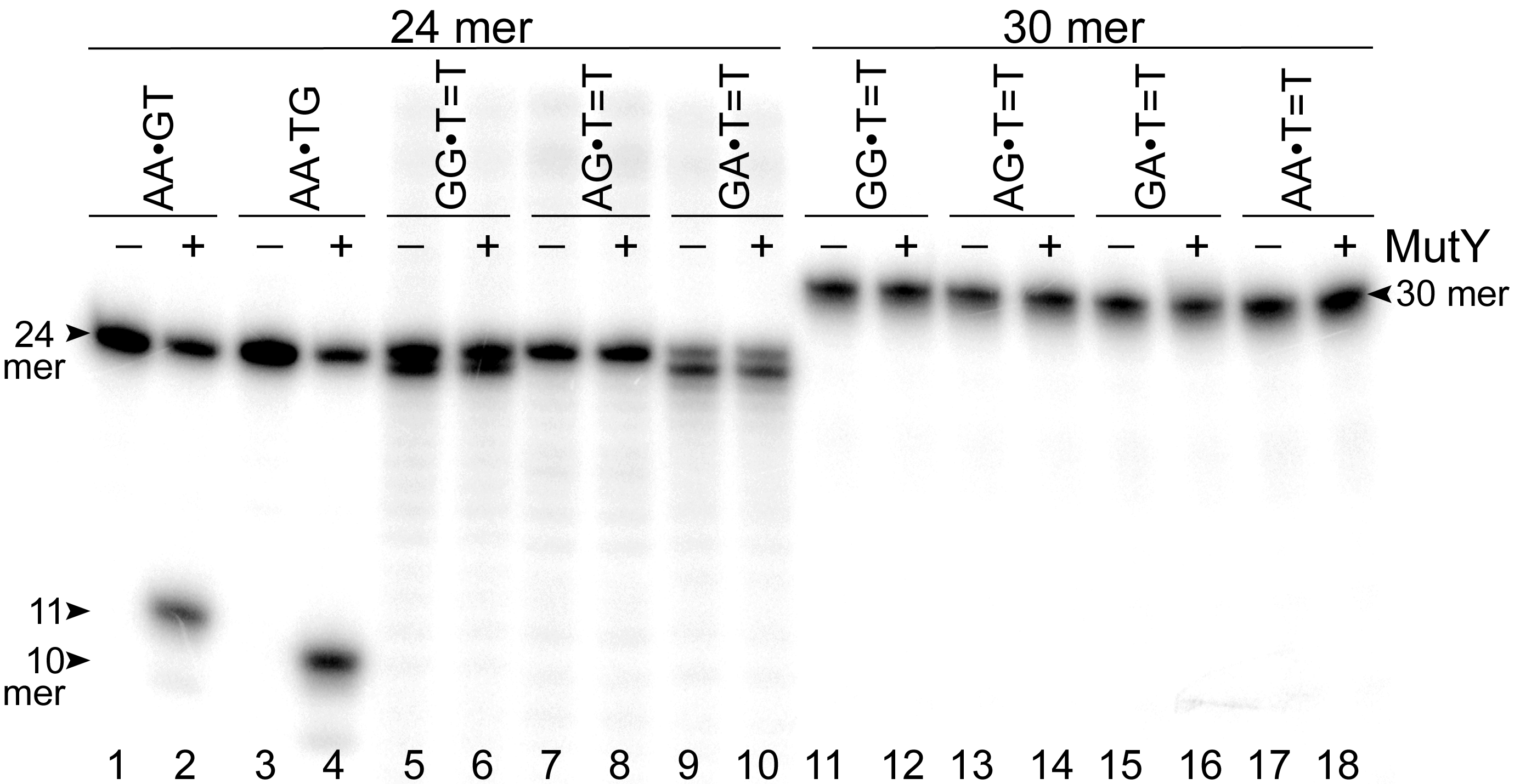

Supplement: Supplemental Information 7 — Lanes 1, 3, 5, 7, 9, 11, 13, 15 and 17, control non-treated 24 and 30 mer duplex oligonucleotides; lanes 2, 4, 6, 8, 10, 12, 14, 16 and 18, 24 and 30 mer duplex oligonucleotides incubated with MutY. For details see materials and Methods. [file peerj-06-6029-s007.png]

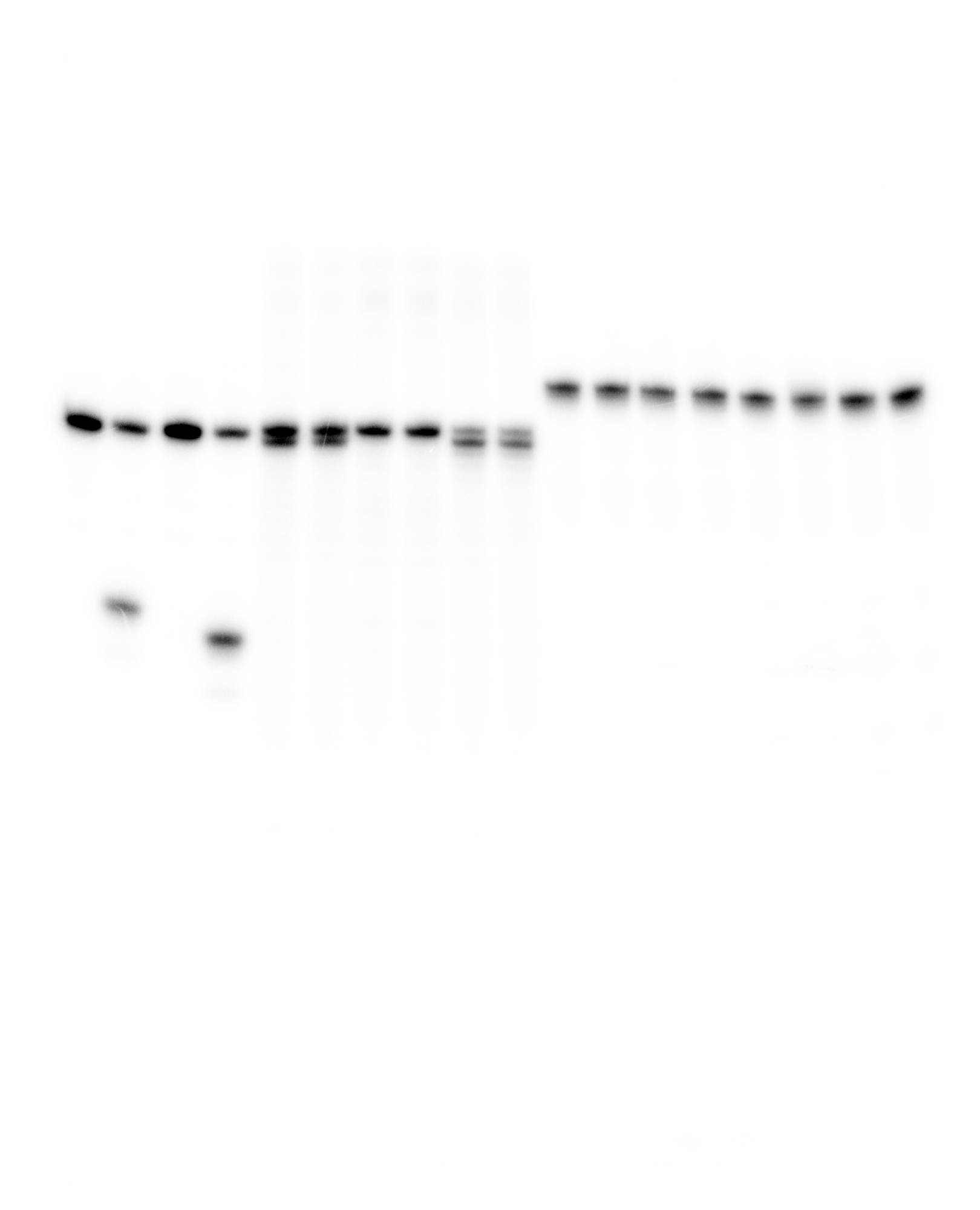

Supplement: Supplemental Information 8 — Analysis of the cleavage products generated by MutY when acting upon 5′-[32 P]-labelled 24 and 30 mer duplex oligonucleotides containing the G• T mismatch and CPD adduct. For details see materials and Methods see Fig. S1. [file peerj-06-6029-s008.png]

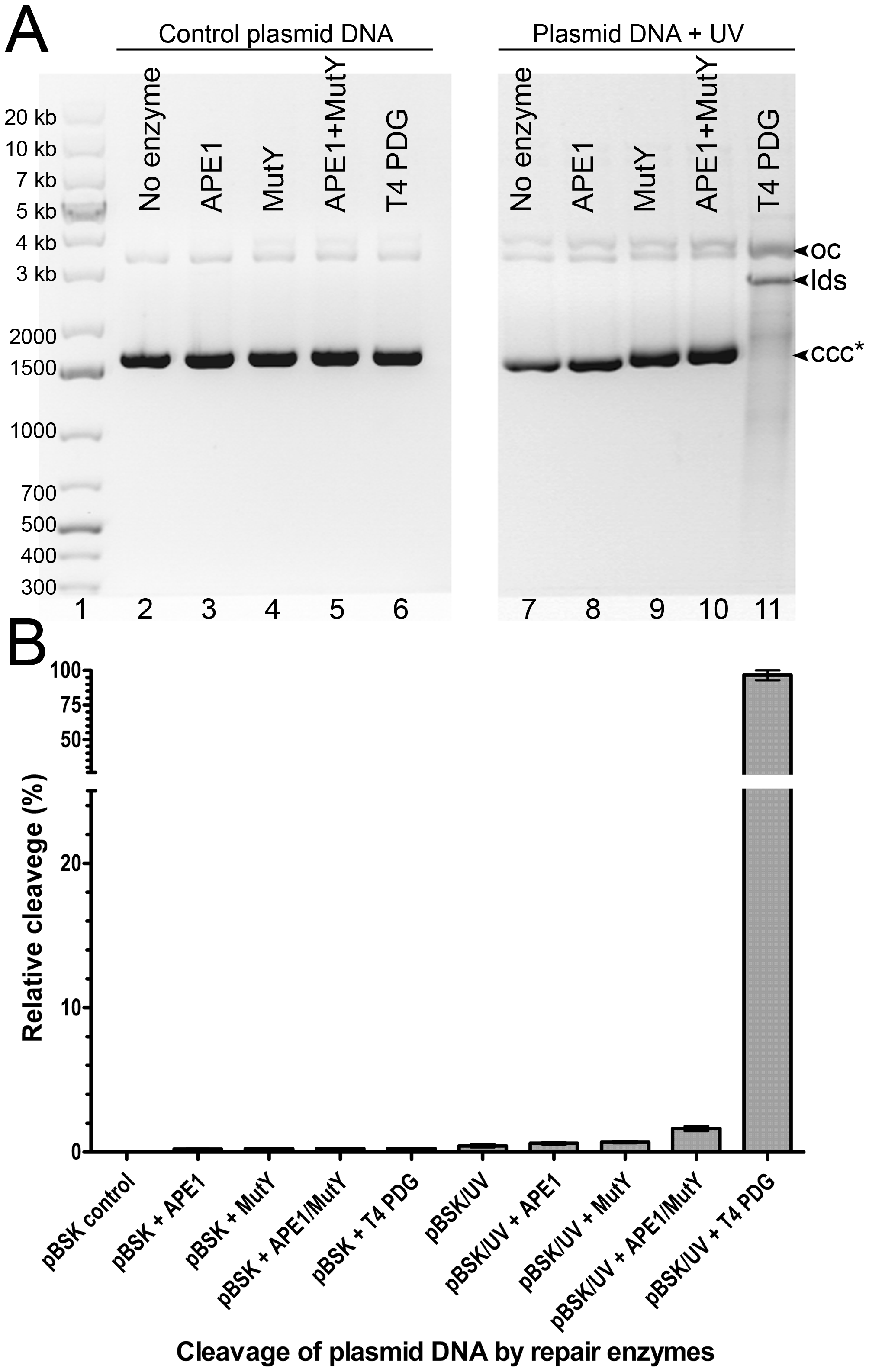

Supplement: Supplemental Information 9 — (A) Agarose gel electrophoresis (0.8%) of the cleavage products generated by MutY, APE1 and T4 PDG when acting upon supercoiled (ccc) form of plasmid DNA. Lane 1, GeneRuler 1 kb DNA ladder; lanes 2–6, control non-treated plasmid DNA; lanes 7–11, UV-irradiated plasmid DNA. The arrows denote the position of “ccc”, “oc” and “lds” forms of plasmid DNA . For details see Materials and Methods. (B) Graphical representation of data from panel A. [file peerj-06-6029-s009.png]

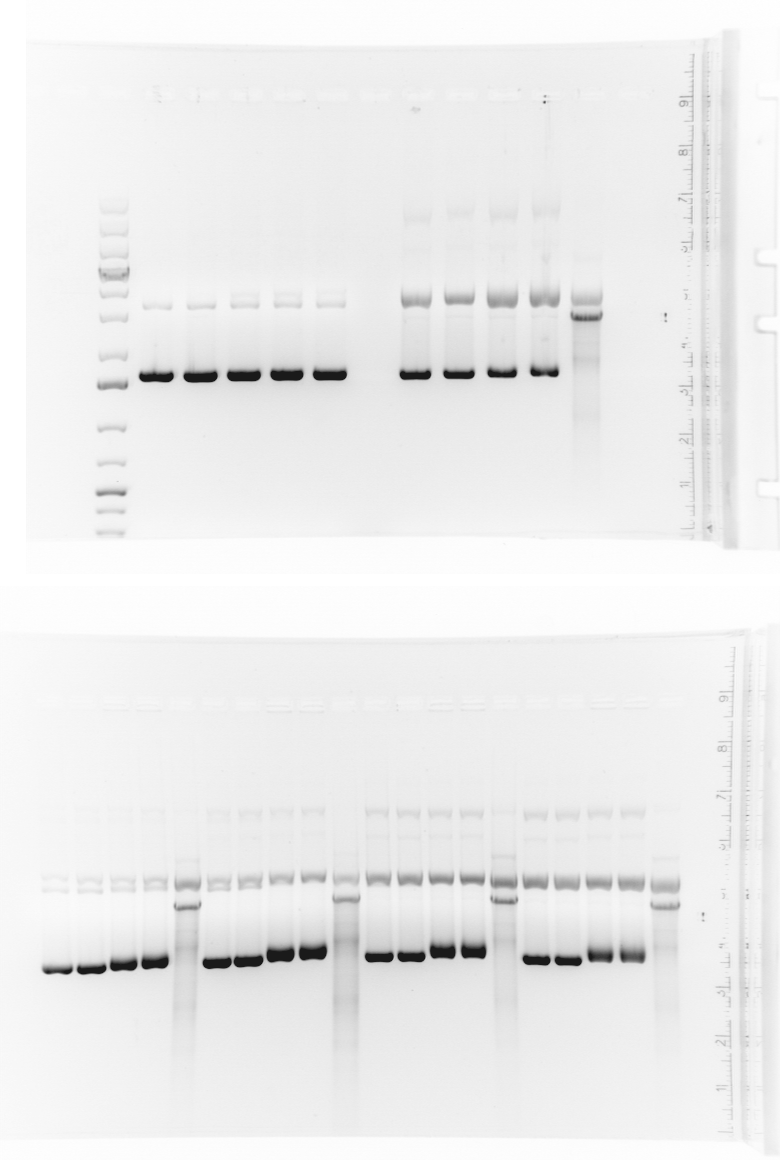

Supplement: Supplemental Information 10 — Cleavage of the UV-irradiated pBlueScript SK(+) plasmid DNA by DNA repair enzymes. (A) Agarose gel electrophoresis (0.8%) of the cleavage products generated by MutY, APE1 and T4 PDG when acting upon supercoiled (ccc) form of plasmid DNA. For details see Fig. S2. [file peerj-06-6029-s010.png]
